# Hematobiochemical, Oxidative Stress, and Histopathological Mediated Toxicity Induced by Nickel Ferrite (NiFe_2_O_4_) Nanoparticles in Rabbits

**DOI:** 10.1155/2022/5066167

**Published:** 2022-03-11

**Authors:** Muhammad Shahid Khan, Saeed Ahmad Buzdar, Riaz Hussain, Gulnaz Afzal, Ghazala Jabeen, Muhammad Arshad Javid, Rehana Iqbal, Zahid Iqbal, Khola Bint Mudassir, Saba Saeed, Abdur Rauf, Hafiz Ishfaq Ahmad

**Affiliations:** ^1^Institute of Physics, The Islamia University, Bahawalpur 63100, Pakistan; ^2^Department of Pathology, Faculty of Veterinary and Animal Sciences, The Islamia University, Bahawalpur 63100, Pakistan; ^3^Department of Zoology (Life sciences), The Islamia University, Bahawalpur 63100, Pakistan; ^4^Department of Zoology, Lahore College for Women University, Lahore, Pakistan; ^5^Department of Basic Sciences, University of Engineering and Technology, Taxila, Pakistan; ^6^Institute of Pure and Applied Biology, Zoology Division, Bhauddin Zakariya University, Multan, Pakistan; ^7^Department of Pharmacology, Faculty of Veterinary and Animal Sciences, The Islamia University, Bahawalpur 63100, Pakistan; ^8^Department of Chemistry, University of Swabi, Swabi-Anbar KPK, Pakistan; ^9^Department of Animal Breeding and Genetics, University of Veterinary and Animal Sciences, Lahore, Pakistan

## Abstract

From the past few decades, attention towards the biological evaluation of nanoparticles (NPs) has increased due to the persistent and extensive application of NPs in various fields, including biomedical science, modern industry, magnetic resonance imaging, and the construction of sensors. Therefore, in the current study, magnetic nickel ferrite (NiFe_2_O_4_) nanoparticles (NFNPs) were synthesized and evaluated for their possible adverse effects in rabbits. The crystallinity of the synthesized NFNPs was confirmed using X-ray diffraction (XRD) technique. The saturation magnetization (46.7 emug^−1^) was measured using vibrating sample magnetometer (VSM) and 0.35-tesla magnetron by magnetic resonance imaging (MRI). The adverse effects of NFNPs on blood biochemistry and histoarchitecture of the liver, kidneys, spleen, brain, and heart of the rabbits were determined. A total of sixteen adult rabbits, healthy and free from any apparent infection, were blindly placed in two groups. The rabbits in group A served as control, while the rabbits in group B received a single dose (via ear vein) of NFNPs for ten days. The blood and visceral tissues were collected from each rabbit at days 5 and 10 of posttreatment. The results on blood and serum biochemistry profile indicated significant variation in hematological and serum biomarkers in NFNP-treated rabbits. The results showed an increased quantity of oxidative stress and depletion of antioxidant enzymes in treated rabbits. Various serum biochemical tests exhibited significantly higher concentrations of different liver function tests, kidney function tests, and cardiac biomarkers. Histopathologically, the liver showed congestion, edema, atrophy, and degeneration of hepatocytes. The kidneys exhibited hemorrhages, atrophy of renal tubule, degeneration, and necrosis of renal tubules, whereas coagulative necrosis, neutrophilic infiltration, and severe myocarditis were seen in different sections of the heart. The brain of the treated rabbits revealed necrosis of neurons, neuron atrophy, and microgliosis. In conclusion, the current study results indicated that the highest concentration of NPs induced adverse effects on multiple tissues of the rabbits.

## 1. Introduction

During the last few decades, application of nanotechnology in different fields like treatment therapy, biotechnology, nutrition, aerospace engineering, and electronics has rapidly grown across the globe. Nanoparticles (NPs) are extensively and persistently investigated for their toxic impacts on the cells, immune tissues, and DNA damages [1, 2]. The increased production and use of NPs has induced different disorders in target- and nontarget-exposed animals including a high risk of respiratory problems and abnormalities in immune functions [3]. Biomedical applications of NPs have tremendously increased due to advancements in technology and innovation in applications like new synthesis procedures and investigation of effectiveness in vivo and in vitro [4, 5]. Among the different types of NPs, ferrite NPs are extensively used as catalysts in various biological reactions, targeted drug delivery, oncological treatments, and targeted therapy and in the storage of small and big data storage devices, sensors, and magnetic-based drug administration [6–8]. Previously, studies indicated that ferrite NPs are used as a diagnostic tool for high saturation magnetization, tissue relaxivity, clarity, and exact localization of these particles in the target tissue [9–12]. It is reported that iron oxide being ferrite in nature is extensively used in various fields of nanotechnology [13–15]. Reports indicate that among different ferrites, nickel and cobalt ferrites are also used to increase the high magnetic moment of ferrites in biomedical applications [16–18]. NFNPs are also used in a variety of research experiments due to their low magnetic anisotropy, thermal stabilities, interesting electrical and magnetic properties, and high bulk saturation magnetization [19] and as a contrast enhancement magnetic NPs in biomedical applications [20, 21]. Moreover, these NPs are commonly used due to high relaxivity and tremendous potential for magnetic resonance imaging [22]. Being a heavy metal, nickel combined with NO3, SO4, CO3, and Cl2 induces adverse toxicological effects on environmental and public health [23, 24]. Different previous studies have investigated the cytotoxic and cancer-inducing effects of nickel. In vitro studies revealed that NPs and their compounds can cause genotoxicity [25] and dysregulation of apoptotic pathways [26, 27]. Oxidative stress occurs due to the overproduction of reactive oxygen species during the impairment of various normal physiological functions and apoptotic pathways [28, 29]. Different studies have determined that Ni NPs induced oxidative stress via increased generation of free radicals [30, 31]. Almost all the NPs are administered intravenously for diagnostic and multiple therapeutic purposes in animals and humans. Therefore, monitoring and investigating possible adverse impacts on various body tissues, including blood, is vital [3, 32]. However, there is a considerable gap of knowledge regarding the exact mechanisms of induction of oxidative stress due to NFNPs in a time- and dose-dependent manner. Previously, no information concerning the toxic effects of NFNPs on brain of treated animal is available. Less data is available regarding the toxicological impacts of NFNPs on hematology, serum biochemistry, oxidative response, and histopathology of visceral tissues of exposed animals. Therefore, for the first time, this study investigated the adverse toxic ailments on blood, serum chemistry, oxidative responses, and histopathology of various visceral organs like brain, kidneys, spleen, liver, and heart of rabbits.

## 2. Material and Methods

### 2.1. Chemicals and Synthesis of Ferrite Nanoparticles

All the chemicals were of analytical grade and obtained from Sigma-Aldrich (USA) and Merck (Germany). Commercial chemicals were procured from Sigma and utilized without any further purification. NFNPs were produced using the coprecipitation technique [33, 34].

### 2.2. Structural Properties with Chracterisation and Evaluation of Ferrite Nanoparticles

#### 2.2.1. X-Ray Diffraction (XRD) Analysis

The X-ray diffraction (XRD) technique was carried out to confirm the crystalline structure of the synthesized nanoparticles. The crystallinity, average particle size, and phase identification of nanoparticles were determined using a Bruker-D8 Advance Laboratory Diffractometer (with CuK*α*1 radiation, *λ* = 1.54 Å). The Scherrer equation (Dhkl = 0.9*λ*/*β* cos *θ*) was employed to investigate the average crystallite size of the produced samples [35, 36].

#### 2.2.2. Transition Electron Microscope (TEM) Analysis

The TEM (transition electron microscope) was used to examine the microstructure morphology of the synthesized NPs. Also, it confirmed the size of the synthesized nanoparticles. A suitable statistical histogram plot was used to determine the mean particle size [37, 38].

#### 2.2.3. Vibrating Sample Magnetometer (VSM) Analysis

The VSM analysis was used to confirm the magnetic behavior of the synthesized material. According to the previous literature [39, 40], the saturation magnetization of the hysteresis loop was investigated using the VSM (Lakeshore 7407).

#### 2.2.4. Magnetic Resonance Imaging (MRI) and Relaxivity Analysis

MRI was used to investigate the contrasting impact of our synthesized samples in rabbit liver and spleen. MRI was also used to examine the intensity of the region of interest in rabbit organs. Relaxivity of the liver and spleen was determined using MRI data [41, 42].

#### 2.2.5. Experimental Animal Treatment Planning

For biological evaluation, a total of 16 rabbits (*Oryctolagus cuniculus*) of the same age and body mass were obtained from a local private market of district Bahawalpur, Punjab Province, Pakistan. Study rabbits were kept under similar housing conditions. All the animals were free from any clinical signs of disease and had free access to normal food and clean water during the trial. The animal house was cleaned and disinfected before the start of the trial. After a few days of adaptation to laboratory conditions, the rabbits were randomly picked, divided, and placed in two groups equally. After that, the rabbits in group B were administered with NFNPs via ear vein, while the rabbits in group A were considered normal control animals. All the experimental rabbits were monitored for clinical and behavioral changes throughout the trial.

#### 2.2.6. Blood and Serum Biochemistry Analyses

Each rabbit from the experimental groups was anesthetized before collection of blood. About 5.0 mL blood was obtained from the vein (tail vein) of each rabbits at days 5 and 10 of postexposure. All the blood samples were immediately processed for hematological and serum biochemistry changes. The hematological profile of each rabbit was determined using an automated hematology analyzer (Sysmex XE-2100, Japan) according to a previous protocol [43, 44]. The serum was removed from each blood sample, and different serum biochemical biomarkers of the liver, kidneys, and heart following were measured using commercial kits with the help of a chemistry analyzer [45].

#### 2.2.7. Antioxidant Enzymes and Oxidative Stress Biomarkers

The quantity of different serum antioxidant enzymes like catalase (CAT), supper oxide dismutase (SOD), reduced glutathione, and oxidative stress parameters such as malondialdehyde concentrations (MDA) were determined with the help of spectrophotometer according to the previous procedure [46].

#### 2.2.8. Gross and Microscopic Observations

For gross and microscopic changes, four rabbit from each group were euthanized after blood collection on days 5 and 10 of the experiment. The heart, brain, liver, spleen, and kidneys were carefully examined, and a small piece of each tissue was fixed in neutral buffered formalin (10%). After a few days of preservation, all the tissues were processed for microscopic observation using standard histological procedures of commonly used hematoxylin and eosin staining [47].

### 2.3. Statistical Analysis

The data on different blood and serum profile was subjected to suitable statistical software for a significant difference. For the pictorial presentation, MRI (RadiAnt DICOM Viewer), XRD (Spec Viewer, Origin pro), VSM (Origin pro), and TEM (ImageJ) software were used.

## 3. Results

### 3.1. XRD and VSM Analysis

**Figure 1:**
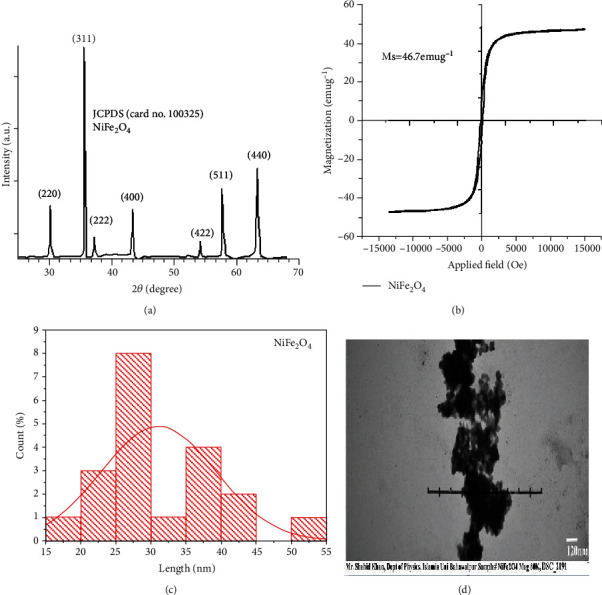
(a) XRD results showing phase identification patterns of NiFe_2_O_4_. (b) VSM plot showing magnetic behavior of NiFe_2_O_4_. (c) Histogram photograph showing average particle size of NiFe_2_O_4_. (d) TEM images of NiFe_2_O_4_.

The results on XRD patterns of NFNPs with prominent peaks having hkl values (220), (311), (222), (400), (422), (511), and (440) planes at 2theta angles of 30.4, 35.9, 37.3, 43.7, 53.9, 57.7, and 63.1 fully matched with JCPDS (File no. 10-0325) are presented in Figure 1(a). The size of synthesized NFNPs using the Scherrer equation *D* = 0.9*λ*/*β* cos *θ* was 30 nm. The structure of synthesized NFNPs by TEM analyses is indicated in Figure 1(d). The size of nanoparticles ranges from 12 nm to 57 nm, with an average of 31.2 nm. The normal fit tool determined their mean particle size, around 15-55 nm (Figure 1(c)). The magnetization curve for synthesized NFNPs measured by VSM is shown in Figure 1(b). The hysteresis loop area was almost zero, and the samples' saturation magnetization (Ms) was 46.7 emug^−1^.

### 3.2. MRI and Relaxivity Analysis

**Figure 2:**
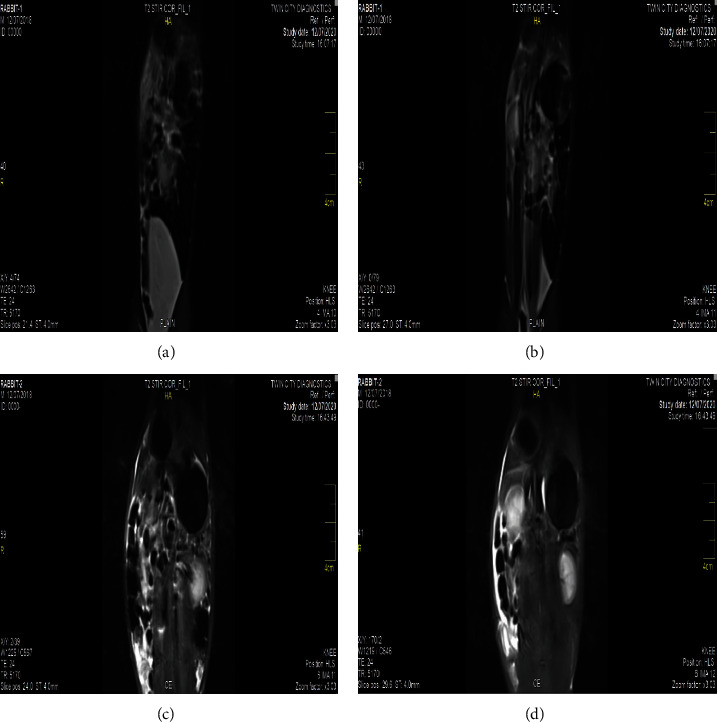
(a and b) MRI Images of liver and spleen of the plain/control rabbits showing no contrast. (c and d) MRI Images of liver and spleen of the treated rabbits showing contrast.

**Figure 3:**
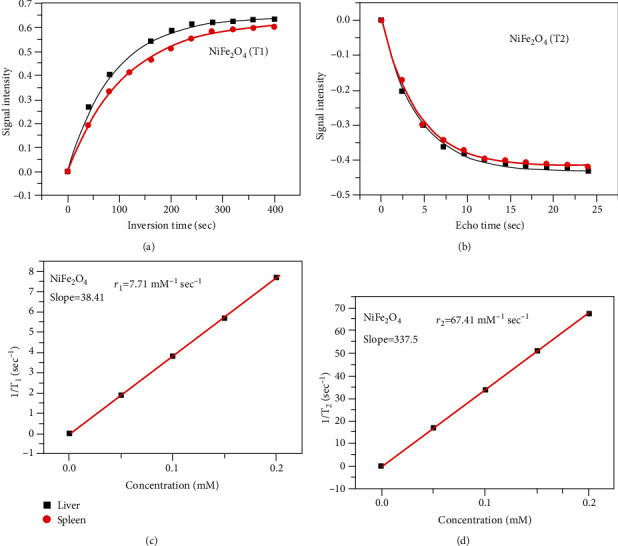
(a) Signal intensity for T_1_ weighted. (b) Signal intensity for T_2_ weighted. (c) Relaxivity r_1_. (d) Relaxivity r_2_.

The result on contrast analysis of NFNPs in normal (Figures 2(a) and 2(b)) and treated rabbits (Figures 2(c) and 2(d)) showed clear contrast with signal strength induced by contrast agent for liver (*I* = 94.2, SD = 28) and spleen (*I* = 61, SD = 14). T1 and T2 relaxivity (Figures 3(a) and 3(b)) is quantified using inversion time (TI) and echo time (TE) as well as signal intensity along the *y*-axis (Figures 3(c) and 3(d)) which depicts the findings of 1/T1 and 1/T2 as a function of concentration. The slopes of the usual lines 1/T1 and 1/T2 were 38.41 mM^−1^ s^−1^and 337.5 mM^−1^ s^−1^, respectively. The corresponding relaxivities r1 and r2 were 7.71 mM^−1^ s^−1^ and 67.42 mM^−1^ s^−1^. At field strength of 0.35 T, the relaxivity ratio r2/r1 for NFNPs contrast agent was 8.744.

### 3.3. Hematological and Serum Analysis

**Figure 4:**
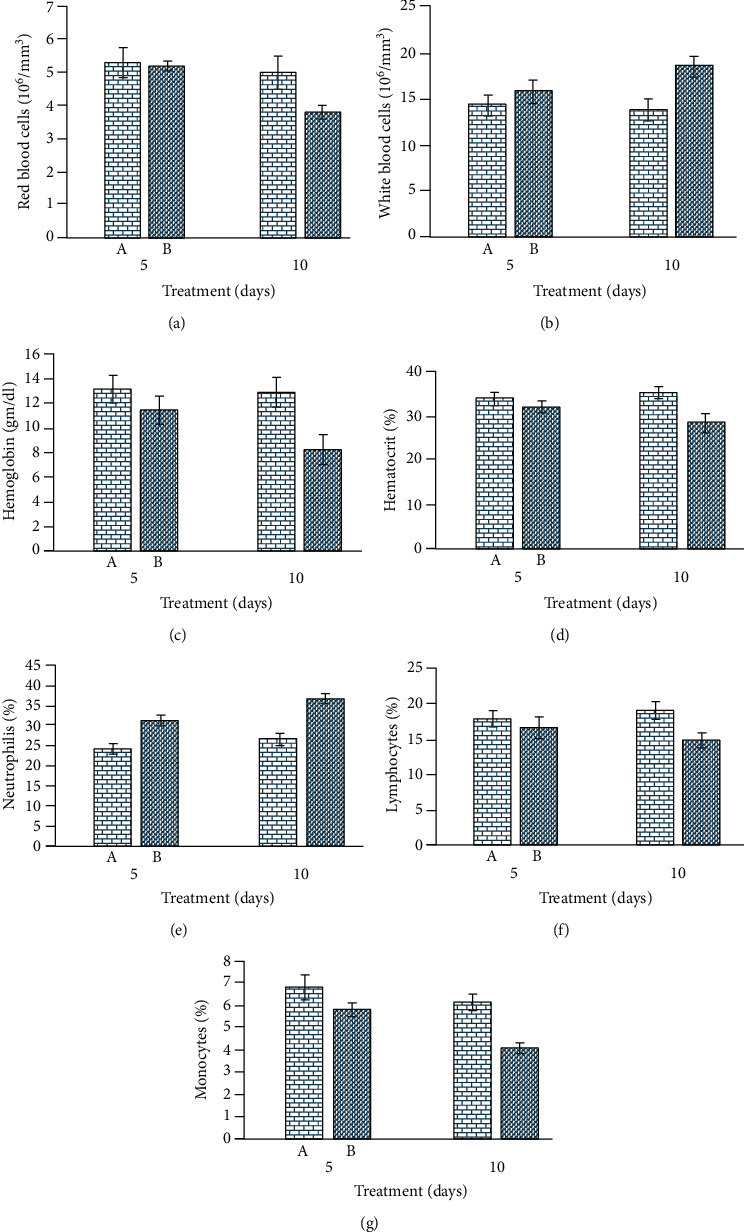
Photograph exhibiting hematological alterations: (a) red blood cell counts; (b) white blood cell counts; (c) hemoglobin quantity; (d) hematocrit percentage; (e) neutrophil counts; (f) lymphocyte counts; and (g) monocyte counts in iron oxide nanoparticle-treated rabbits.

**Figure 5:**
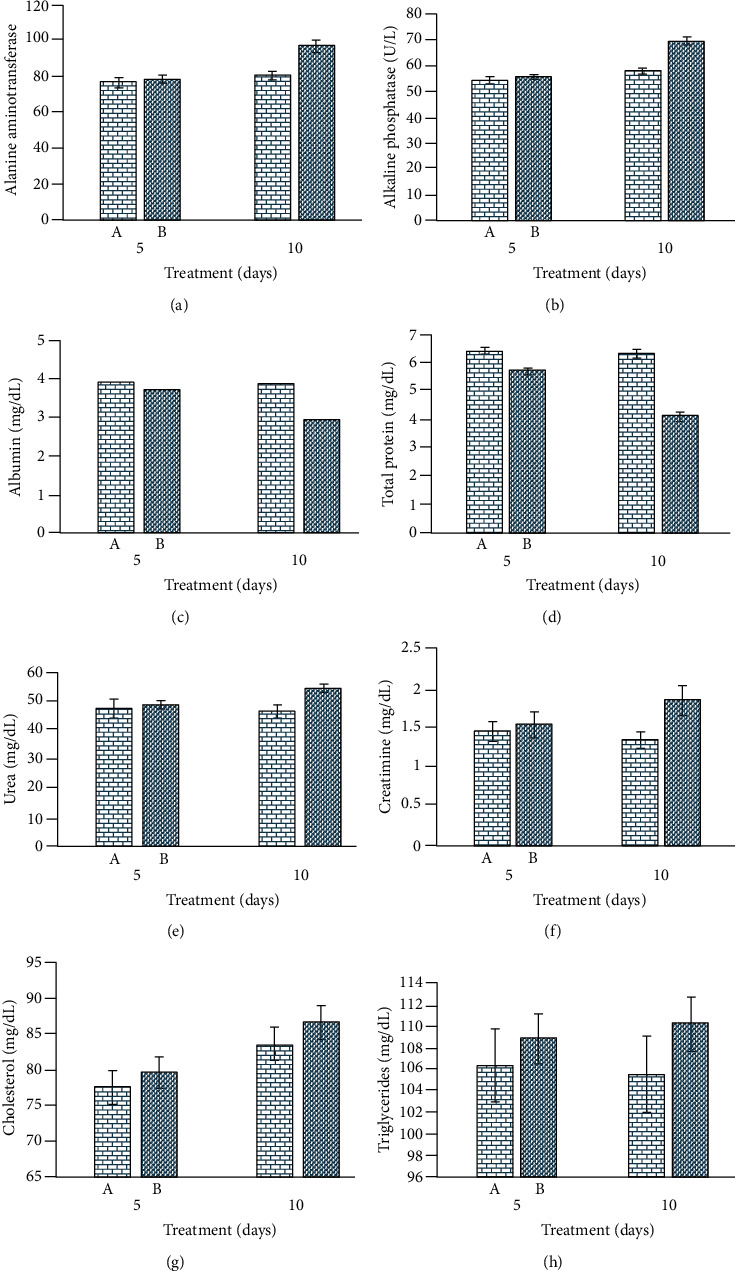
Photograph exhibiting serum biochemical profile; (a) alanine aminotransferase; (b) alkaline phosphatase; (c) albumin; (d) serum total proteins; (e) urea; (f) creatinine; (g) cholesterol; and (h) triglyceride in iron oxide nanoparticle-treated rabbits.

In treated rabbits, different blood parameters (Figure 4) like red blood cell, monocyte, lymphocyte, hemoglobin, and pack cell volume were significantly decreased at days 5 and 10 of posttreatment. The values of total leukocyte and neutrophil cells increased in treated rabbits. The results on different serum biochemical investigations in treated rabbits, including total serum proteins and albumin quantity, decreased significantly at days 5 and 10 of postexposure. The values of serum enzymes like aspartate aminotransferase, alanine aminotransferase, and alkaline phosphatase, along with serum bilirubin, were significantly increased at both experimental days. The values of renal function tests (urea and creatine) were significantly higher in exposed rabbits. In treated rabbits, the quantity of serum cholesterol, glucose, and triglycerides was significantly higher (Figure 5).

### 3.4. Antioxidant Enzymes and Oxidative Stress Parameters

**Figure 6:**
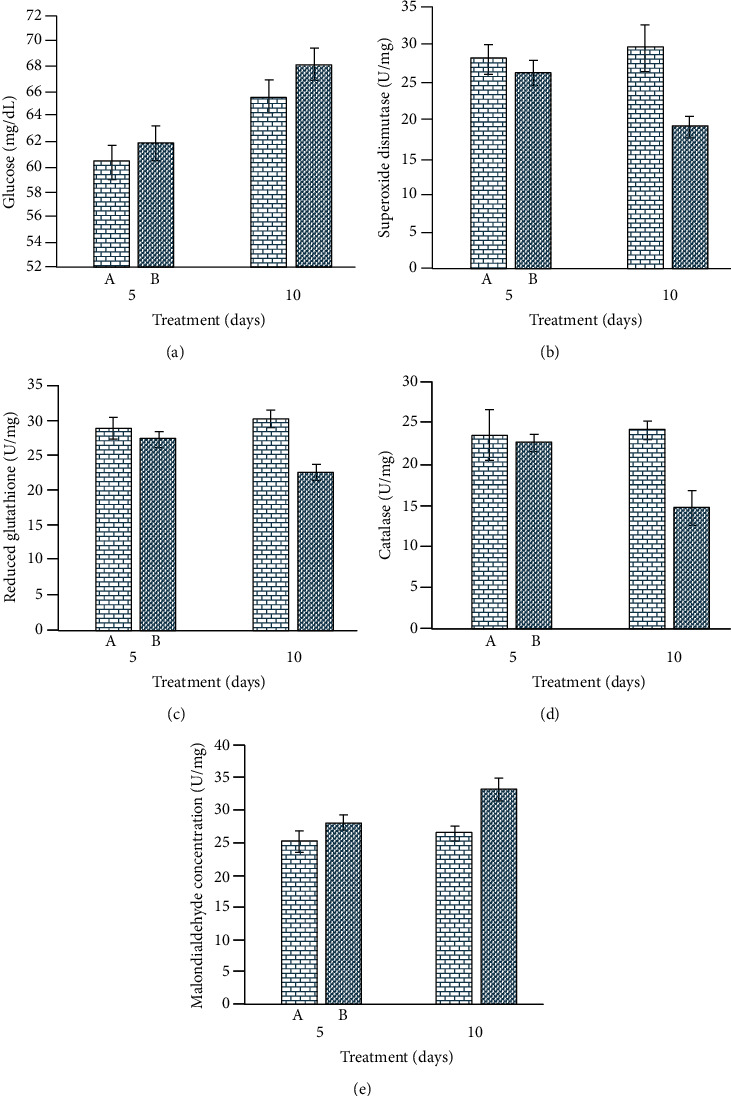
Photograph showing changes in (a) serum glucose; (b) superoxide dismutase oxidative; (c) reduced glutathione; (d) catalase; and (e) malondialdehyde concentrations in iron oxide nanoparticle-treated rabbits.

The quantity of different antioxidant enzymes, including superoxide dismutase, peroxidase, reduced glutathione, catalase, and malondialdehyde concentration, showed significantly lower values in nanoparticle-treated rabbits than untreated control rabbits. The values of oxidative stress parameters (malondialdehyde) were significantly higher in treated rabbits on day 10 of the trial (Figure 6).

### 3.5. Microscopic Observations

**Figure 7:**
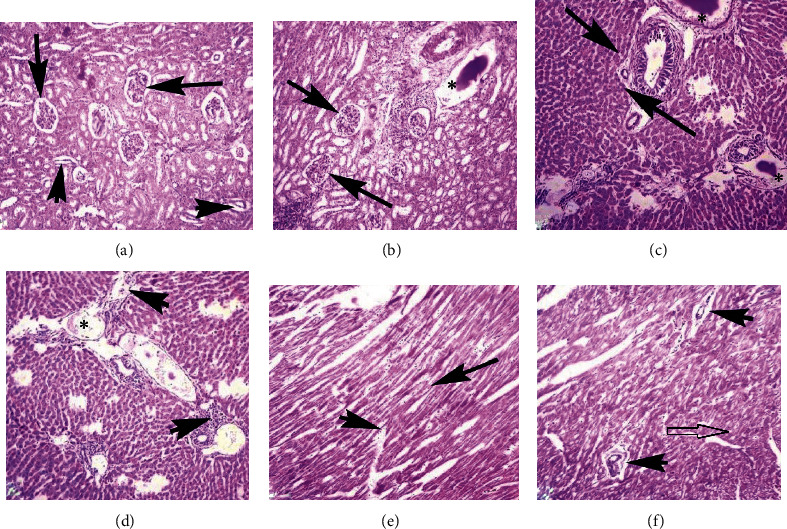
Photomicrograph showing necrosis of renal tubular epithelium, increased urinary space (arrows), degeneration of renal tubules and disorganization of glomeruli (arrowheads), and edema (∗) in the kidneys (a and b); degeneration and pyknosis of hepatocyte, edema (∗), fatty change (arrow), and inflammatory materials (arrowheads) in various sections of the liver (c and d); and coagulative necrosis (arrow), inflammatory exudate, disruption of cardiac muscles (arrowheads), and vacuolar degeneration (empty arrow) in heart sections of the rabbits (e and f) at day 10 of exposure (400x, H&E stain).

**Figure 8:**
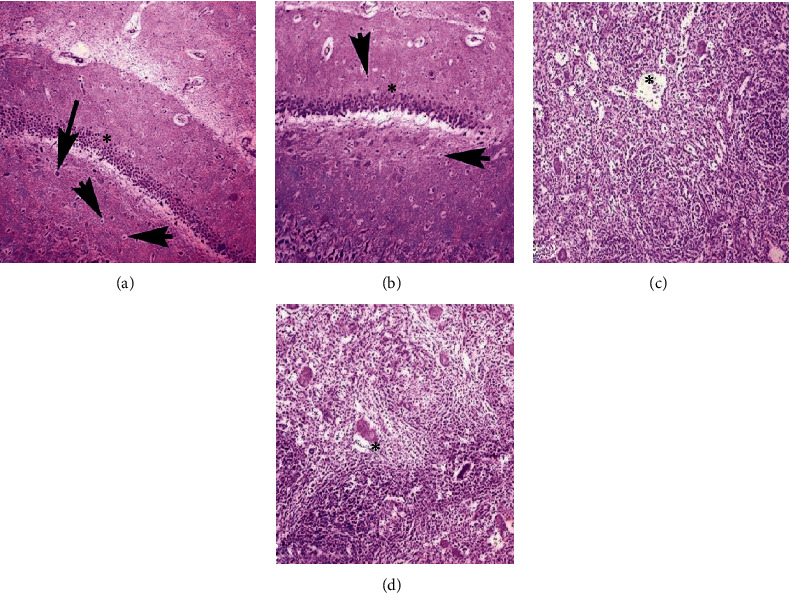
Photomicrograph showing atrophy (arrow heads) and necrosis of neuron (arrow), microgliosis (∗), and congestion in the brain of treated rabbits (a and b). Inflammatory exudate (∗) along with depletion, degeneration of red and white pulp, and depletion of lymphoid tissues (c and d) at day 10 of exposure (400x, H&E stain).

At the necropsy level, no gross ailments were observed in the visceral organs of control and treated rabbits. Microscopic observation of kidneys of nanoparticle-treated rabbits did not exhibit histological alterations at day 5 of posttreatment. However, different microscopic abnormalities in the kidneys of rabbits at day 10 of postexposure tissue like severe necrosis, hemorrhages, congestion, widening of urinary space, atrophy of renal tubule, degeneration of glomeruli, and necrosis of renal tubules were observed. Different histological alterations like necrosis of hepatocyte, congestion, edema, atrophy, and degeneration of hepatocyte and congestion were observed in liver sections of treated rabbits at day 10 of exposure. At the microscopic level, various sections of the heart of treated rabbits exhibited congestion, coagulative necrosis, edema, neutrophilic infiltration, severe myocarditis, and degeneration of cardiac myocytes (Figure 7). Microscopic observation of the brain of treated rabbits exhibited different ailments like necrosis of neurons, atrophy of neurons, microgliosis, cytoplasmic vacuolization, and congestion at day 10 of posttreatment. Various microscopic alterations in the spleen of treated rabbits, like degeneration of white and red pulp and depletion of lymphoid, were examined (Figure 8).

## 4. Discussion

Due to the widespread use of nanoparticles in biomedical and biological sciences, the studies have been focused on the monitoring of deleterious impacts of nanoparticles both on the environment and public health [48, 49]. Due to their various magnetic properties like biodegradability and biocompatibility, NFNPs play a crucial and important role in treatment therapy in a variety of biomedical fields such as treatment of cancer, cellular therapy, and tissue repairing [9]. Hence, this study was designed to determine possible adverse effects on the blood, serum biochemistry, and various visceral tissues of rabbits. Prior to the experimental trial, the NFNPs were synthesized and confirmed. The XRD pattern of synthesized NFNPs, scattering angles, and their corresponding hkl values entirely matched with JCPDS File no. 10-0325 [50]. Small particle size and some pours revealed a large surface area of nanoparticles with strong crystallinity [51]. The TEM images demonstrated the excellent lattice fringes in specific regions of an individual particle, which also supported the development of fine-quality crystalline nanoparticles [52]. The super magnetic behavior of NFNPs was validated by the nearly zero value of the hysteresis loop area. As previously reported, NFNPs can be employed for biomedical applications such as MRI contrast enhancement [53, 54] due to their high saturation magnetization (Ms) of 46.7 emug^−1^. Further doping the metals such as nickel and others can be used to increase the further high magnetic moment of ferrites [16].

As a consequence, the proposed NFNPs could be used as a negative contrast in diagnostic testing to identify organ pathology even in MRI equipment with specialized fields [55]. NFNPs with these qualities are better for in vivo biological applications such as MRI contrast agents and relaxivity, according to the published studies [56, 57]. In the study of MRI contrast enhancement, nickel ferrites can be used as theranostic agents in diagnostic and therapeutic applications [58]. The signal intensity caused by contrast agents such as NFNPs was assessed using IQ View software. The relaxivity ratio r2/r1 is employed in contrast agent selection since it may be increased or reduced based on the size, charge concentration, and field intensity. Based on relaxivity, T1 relaxation appears to be somewhat faster in the liver than in the spleen, and T2 relaxation appears to be similarly a little faster in the liver than in the spleen. Previous studies, on the other hand, have discovered varying relaxation rates in the liver and spleens of rats and rabbits. As a result, the proposed NFNPs might be used as a negative contrast in diagnostic imaging to detect organ illness, even on low-field MRI equipment. According to the published research, NFNPs with these characteristics are ideal for in vivo biomedical applications like MRI contrast agents and relaxivity [59, 60].

In our experimental study, various blood parameters, including lymphocytes, monocytes, hemoglobin, and red blood cells, significantly reduced, while white blood cells and neutrophils significantly increased. The lower hematological parameters in NFNP-treated rabbits could be due to the deleterious effects of NFNPs on blood-forming tissues. The lower hematological profile could also be related to the induction of oxidative stress on bone marrow. The increased total white blood cells and neutrophils can be due to injuries in association with the increased and rapid generation of free radicals in treated rabbits [61]. The significant change in hematological parameters could also be related to the toxic effect of NFNPs on kidneys. It has been recorded that hematological and biochemical parameter can be used as useful and reliable bioindicators in different animals are well-known targeted organs of toxicity after exposure to xenobiotic [45]. In contrast to our hematological investigations, an increased percentage of monocyte, total number of white blood cells, and neutrophil percentage have also been reported in rats [62, 63]. The hematological disorder in treated rabbits might be the entrance of NFNPs in bone marrow tissues due to their small size. Previously, the cytotoxic effects of NPs have also been observed. The lower values of red blood cells could also be due to the binding of NFNPs to the surface of red blood cells leading to hemolysis [64, 65]. In addition, the toxic effects of NPs on bone marrow cells have also been reported [66]. Different published reports showed that the possible toxic effects of nanoparticles depend upon numerous factors like chemical composition, breakdown in the body, retaining time in tissues, dosage, size, bioavailability, immunogenicity, removal from the body, organ specific toxicity, pharmacokinetics, biodistribution, and specific characteristics (surface to volume ratio) in exposed animals [67, 68]. It is recorded that nanoparticles induce their toxic effects via arrest of growth induction of inflammation, stimulation of reactions in defensive responses, and triggering of various neurobehavioral in different cell lines in treated animals [69]. Furthermore, the magnetic nanoparticles after entering in the tissues and different cells potentially affect the nuclear events and damage to cell membranes ultimately leading to blockage or leakage of cytoplasmic contents and disruption of normal metabolic activity [70]. In addition, studies have determined that nanoparticle's influence with physiological metabolism of iron inhibit cellular proliferation and normal cell cycle and induce genotoxicity via damage to various components of proteins and DNA material in the cells.

In the present study, significantly increased concentrations of different serum biochemical biomarkers, including serum enzymes like aspartate aminotransferase, alanine aminotransferase, and alkaline phosphatase along with serum bilirubin, were significantly increased in treated rabbits. The increased concentration of liver function test, kidney function test, and different other serum parameters in treated rabbits could be due to the toxic effects of NFNPs on the liver, kidney, and different other tissues of the body. The significantly increased concentration of serum biochemistry parameters might be due to oxidative stress in the liver and kidneys in association with induction of tumor necrosis factor in NFNPs [71]. Previously, increased concentrations of superoxide dismutase, blood urea nitrogen, and MDA in various tissues (kidneys, liver, and heart) while lower values of catalase in kidneys and heart and serum total proteins along with albumin in mice were estimated due to nickel oxide NPs. Similarly, significant variations in hematological (Hb content, RBC, and WBC count), enzymological (ALT, AST, LDH, and ALP), and biochemical [72–74]. In contrast to our results on oxidative stress and status of antioxidant enzymes, increased values of catalase and glutathione-S-transferase in NiO nanoparticle-treated rats have been recorded [75].

In the published literature, scanty information is available about the microscopic changes in rabbits due to nickel ferrite nanoparticles. The microscopic changes in our study in rabbits might be due to the rapid depletion of antioxidant enzymes (as evident in this study) and increased amount of reactive oxygen species (ROS), leading to an increased process of lipid peroxidation (oxidative stress) induced by NFNPs. However, different earlier studies have observed various histopathological alterations in the liver (increased liver weight, binucleated hepatocytes, increased akaryotic hepatocytes, hepatic sinus disappearance, cellular edema, and focal areas of necrosis) in rats treated with oxidic nickel nanoparticles [76, 77]. These histopathological changes in the liver and other visceral tissues might be due to the accumulation of nickel ferrite nanoparticles [76, 78]. Furthermore, different histopathological changes in kidneys and spleen of rats exposed to higher doses of oxidic nickel nanoparticles have also been observed [76, 78]. In contrast to our results, no histopathological changes in the spleen, heart, brain, and kidneys were reported in Wistar rats exposed to nickel-containing nanoparticles [79]. Moreover, various microscopic changes in the brain of rats due to nickel oxide nanoparticles like gliosis, necrosis, spongy changes, and hyperemia were observed [78].

## 5. Conclusion

NiFe2O4 nanoparticles with a size of about 30 nm were synthesized by the coprecipitation method. XRD characterization showed exact confirmation of crystallinity and its phase identification. VSM evaluated magnetic behavior. Due to high saturation magnetization value of 46.7 emug^−1^ of synthesized material, it is suggested that these particles can be used in MRI as a contrast agent. Different characteristics of NFNPs such as drug delivery, MRI, and relaxivity are important tools for in vivo biological applications. Furthermore, the results on the biological evaluation of nanoparticles indicated that nickel-coated iron oxide nanoparticles induce adverse effects on hematology, serum biochemistry, oxidative responses, and microscopic alterations in multiple visceral tissues of rabbits.

## Data Availability

The data of the current experiment is part of a PhD thesis and can be obtained from the first author when needed.
